# Characterization and Assessment of a Novel Plate and Frame MD Module for Single Pass Wastewater Concentration–FEED Gap Air Gap Membrane Distillation

**DOI:** 10.3390/membranes9090118

**Published:** 2019-09-06

**Authors:** Rebecca Schwantes, Jakob Seger, Lorenz Bauer, Dniel Winter, Tobias Hogen, Joachim Koschikowski, Sven-Uwe Geißen

**Affiliations:** 1 SolarSpring GmbH, 79114 Freiburg, Germany; 2 Fraunhofer Institute for Solar Energy Systems, 79110 Freiburg, Germany; 3 Institute of Power Engineering, Technische Universität Dresden, 01062 Dresden, Germany; 4 Department of Environmental Technology, Technische Universität Berlin, 10623 Berlin, Germany

**Keywords:** distillation, high recovery rate, brine concentration, zero liquid discharge, membrane distillation module, wastewater concentration, resource recovery

## Abstract

Membrane distillation (MD) is an up and coming technology for concentration and separation on the verge of reaching commercialization. One of the remaining boundaries is the lack of available full-scale MD modules and systems suitable to meet the requirements of potential industrial applications. In this work a new type of feed gap air gap MD (FGAGMD) plate and frame module is introduced, designed and characterized with tap water and NaCl–H_2_O solution. The main feature of the new channel configuration is the separation of the heating and cooling channel from the feed channel, enabling a very high recovery ratio in a single pass. Key performance indicators (KPIs) such as flux, gained output ratio (GOR), recovery ratio and thermal efficiency are used to analyze the performance of the novel module concept within this work. A recovery rate of 93% was reached with tap water and between 32–53% with salt solutions ranging between 117 and 214 g NaCl/kg solution with this particular prototype module. Other than recovery ratio, the KPIs of the FGAGMD are similar to those of an air gap membrane distillation (AGMD) channel configuration. From the experimental results, furthermore, a new MD KPI was defined as the ratio of heating and cooling flow to feed flow. This R_F_ ratio can be used for optimization of the module design and efficiency.

## 1. Introduction

Membrane distillation (MD) has drawn increasing interest in the last 10 years from both academia and applicational parties. Beginning with a wide range of basic testing of various solutions [1,2,3,4,5,6] and the establishment of thermodynamic process understanding and modelling [7,8,9], the state of research has gradually shifted towards a more applied focus, bringing forth more advances in fields of bench and pilot scale trials, membrane testing [10,11] and investigations on membrane hydrophobicity as well as the regeneration of such [12,13,14,15,16,17,18,19]. An increase in modelling, targeted on module and system or even hybrid system scenarios [20,21] together with tecno-economic calculations for industrial applications [22,23] can also be observed. Overall, a shift of perception is gradually taking place in which MD is no longer seen as strongly as a possible substitute for seawater reverse osmosis (SWRO), but its potential as a process step in brine or wastewater concentration and recycling is being unfolded; this is especially valid in cases where reverse osmosis (RO) reaches its functional boundaries due to high osmotic pressure differences. MD is also being adopted in other applications e.g., in the pharmaceutical industry or food industry because it can be operated at ambient pressure and with low temperatures [24,25,26]. 

Despite these increasingly practical advances, MD is not fully commercialized yet and some of the reasons repeatedly given are the lack of full-scale MD modules, high specific thermal energy consumption, low recovery rate and a limited variety of available specialized membranes [27,28]. For example, the regeneration of membrane hydrophobicity in situ and the avoidance of membrane wetting altogether has not yet brought forth a universal strategy for commercial implementation in flat sheet MD modules on a large scale, especially when dealing with high salinity feed solutions—although increasingly targeted by research in the last few years [29,30]. However, promising results have been achieved for specific applications using brines between 1–19% and using porous fluorosiloxane-coated polypropylene hollow fibers [31,32]. 

In addition, technology requirements in industrial applications can be different than those present during the technology development conducted at a time when solar-powered seawater desalination was still the main goal of many MD researchers and developers. With this in mind, and based on the previous experience, new adaptations to MD modules and systems are necessary in conjunction with piloting in the relevant industry. 

Such an opportunity was created by the HighCon project, placed within the funding program WavE–“Future-oriented Technologies and Concepts to Increase Water Availability by Water Reuse and Desalination” [30]. The program was launched by the German Federal Ministry of Education and Research in order to facilitate the adoption of more comprehensive and ecofriendly wastewater treatment and internal reuse of resources in the industry. The project is coordinated by the Technische Universität Berlin, Germany. Within HighCon, the goal is to recover resources from concentrates generated by recycling of industrial wastewater at two different pilot sites. Membrane distillation was represented by the company SolarSpring GmbH and the Fraunhofer Institute for Solar Energy Systems within the consortium. During the project, a zero liquid discharge process chain was developed and piloted, based on a chain of membrane technologies and a humidification-dehumidification (HDH) crystallization step as the final stage. 

Due to the necessity to concentrate the pretreated saline wastewater to a near saturation concentration level in the MD system for subsequent crystallization, a redesign of the MD module and system was carried out and then put into practice in a full-scale prototype. The three main goals to be achieved with the new concept are:

(I) Implementing a plate and frame module design with replaceable inner parts;
(II) Minimization of the number of components in contact with the highly corrosive feed solution;
(III) Enabling of a single pass recovery rate high enough to operate the MD stage as a one- step process.

These design criteria apply to almost any industrial process in which highly corrosive or chemically aggressive or toxic compounds are present in the feed solution and the concentration increase should be high. So far, in the existing MD channel configurations that are based on sensible heat recovery and using flat sheet membranes, the unification of points (I)–(III) were not possible on a full scale and is considered a drawback of the technology. Also, the replacement of inner parts is not possible per se in any module type in which a resin potting is used for sealing of the material layers. Low module recovery rates also mean that to achieve a high increase in concentration, the feed must be circulated in a batch mode or processed by a serial cascade of modules [33]. This requires commercially available heat exchangers and pumps that are chemically resistant to the feed solution to be treated. In this work a new module concept is presented under the name of feed gap air gap membrane distillation (FGAGMD) which incorporates the solution to requirements (I)–(III). Fundamentally, it is a derivation of air gap membrane distillation (AGMD) [34,35], however the feed and the permeate solution are separated from the heating and cooling solution by addition of an extra channel. Precise details on the configuration are provided in Section 2.1 and Section 2.2. Furthermore, the new FGAGMD module was constructed and characterized within the HighCon project. A comprehensive description on the pilot system set-up and experimental configuration is given in Section 2.3. Full characterization data for tap water and artificial NaCl–H_2_O solution with a salinity up to 214 g NaCl/kg is provided in this work and discussed in the results Section 3.

Regarding the topic of specific thermal energy consumption in MD as such, there is still a lack of consensus in publications to provide the key performance indicators necessary to properly evaluate the entire performance of the process. As explained in [22], experiments carried out in small-scale bench testing, are often presented using flux values without any mention of energy consumption [33], thus giving a misleading impression on how future process optimization needs to be carried out. Simply targeting a high gained output ratio (GOR) in MD module design will, however, not satisfy the future market demands, as it is by nature of the process always a trade-off with transmembrane flux, the latter reducing with increasing GOR [36,37,38]. Thus, developments in MD module and process design should be carried out keeping both in mind as described by [22,38]. The question if to maximize GOR or flux should be evaluated carefully on a case to case basis when it comes to the implementation in the industry. Within this work flux, GOR, recovery ratio and thermal efficiency are used to gain a comprehensive understanding of the module performance. 

## 2. Materials and Methods 

### 2.1. Membrane Distillation (MD) Process and the Evolution of Channel Configurations

Membrane distillation is a thermal driven membrane process in which a hydrophobic, microporous membrane constitutes the vapour space. Only vapours can pass through the membranes, non-volatile substances are rejected. The driving force of the process is the effective vapour pressure difference across the membrane most commonly provided by a temperature difference. Mass transfer in MD is well investigated [9,39] and can be described as a combination of molecular and Knudsen diffusion transport mechanisms. Heat transfer in MD is characterized by a combination of latent heat and conductive heat transfer and is sensitive to many parameters such as physical membrane properties, spacer selection and operational parameters [40]. Since the early days of MD [41], the possible MD channel configurations and process designs have evolved in several different directions [42,43,44,45]. One type of MD system, commercially established by the company memsys [46], is a combined thermal and vacuum driven process named VMEMD [47]. Essentially, it represents the process commonly known as multi-effect distillation (MED) equivalent of MD. In this variant the latent heat is recovered and passed on from stage to stage. When talking about plate and frame modules in this work however, the focus will be on the MD process variants in which sensible heat is recovered to increase the efficiency, thus representing a multi-stage flash (MSF) type of heat recovery principle if thought of in terms of state-of-the-art evaporation technology. 

**Figure 1:**
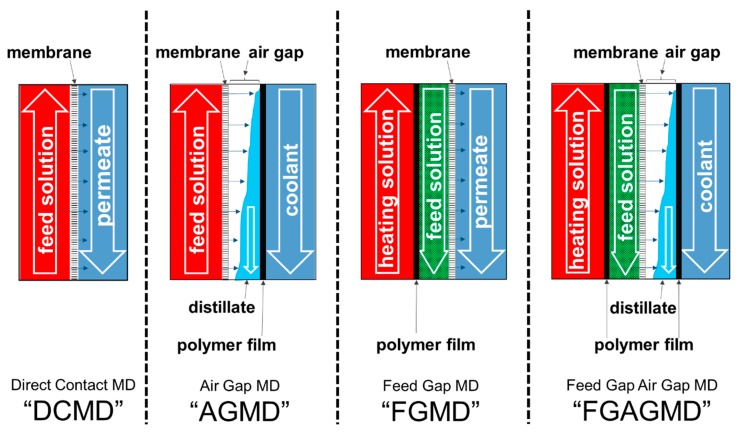
Basic channel configurations direct contact membrane distillation (DCMD) and air gap membrane distillation (AGMD) and derived “Feed gap” variant feed gap membrane distillation (FGMD) and feed gap air gap MD (FGAGMD).

In the following, new derivations of MD channel configurations are introduced. An overview of the commonly known configurations direct contact membrane distillation (DCMD) and AGMD [48], as well as the new configurations feed gap membrane distillation (FGMD) and FGAGMD is provided in Figure 1. 

The DCMD is the simplest MD configuration in which a hot feed solution flows on one side and the cooled permeate flows on the other side of the microporous membrane in counter current. Due to the temperature-induced vapour pressure gradient, vapour is transported through the membrane pores from the hot to the cold side of the membrane which then condenses on entering the permeate stream. An advantage of this configuration is the high flux due to the low thermal barrier of only the membrane material enabling high flux and thermal efficiency at low salinities [36]. However, the fraction of conductive heat passed through the membrane is also comparably higher leading to a potentially lower thermal efficiency in modules with significant heat recovery and especially at higher salinities above 100 g NaCl/kg. There is enough evidence in research to acknowledge that the quality of distillate can, furthermore, be compromised by so called wetting phenomena and that these phenomena are enhanced when there is liquid on both sides of the membrane [14,49]. In DCMD this is unavoidable per se und must, therefore, be considered a disadvantage. Another system-related drawback is the requirement of an additional heat exchanger in order to recover heat from the condenser outlet for pre-heating of the feed. In summary, DCMD is very popular in academia for its simplicity and high flux, but has limitations when it comes to use in the industry and is not as commonly applied on a full scale as e.g., AGMD or PGMD [50,51,52,53,54]. 

The configuration second from the left in Figure 1 is AGMD. In this variant, a thin polymer film separates the distillate channel from the coolant. This also enables the direct internal recovery of heat inside the module, since the feed solution can thereby be used as coolant without. The additional air gap in AGMD also provides a higher thermal insulation between the channels. This reduces the overall heat transfer, thus lowering the flux compared to DCMD but it also reduces the fraction of conductive heat transfer which is not used for evaporation. A comprehensive comparison of the two variants DCMD and AGMD is given by [48]. At higher salinities, DCMD can be more sensitive to the impact of vapour pressure reduction [22] unless thicker membranes are used in which case the thicker membrane substitutes the thermal insulation properties of the air gap [55]. The AG channel variant also gives the possibility of applying a vacuum (v-AGMD) to reduce molecular diffusion resistances or to use low-pressure air sparging to ensure a complete drainage of the distillate, the positive effects of which on wetting are reported in [49]. 

One feature of both DCMD and AGMD are limitations regarding the recovery ratio of distillate in a single pass through the module. Since the thermal energy for evaporation is carried into the module via the feed stream on the evaporator side, the permeate output is limited by the sensible heat contained in that stream. The maximum theoretical recovery ratio in a single pass can be calculated by dividing the latent heat of evaporation at the mean process temperature by the sensible heat supplied be the heating channel. As presented in detail by [38], only approximately 1–8% of the evaporator inlet mass flow can be extracted as permeate in the aforementioned channel configurations. For example, to achieve a concentration increase from 8% *w*/*w* to ~20% *w*/*w* of NaCl H_2_O solution, a recovery ratio of 60% would be necessary in order to reach this goal in a single pass. In order to decouple the thermal energy supply from the feed stream and overcome the limitation of recovery ratio, one possibility is an alternative operation of an AGMD channel set-up. In this so- called FGMD configuration, the feed solution is circulated through the previous air gap and the heating solution is introduced into the former coolant channel which is separated from the feed solution by the polymer film. On the cold side of the membrane, the permeate is now circulated as coolant and the vapours condense in that coolant channel in analogy to the DCMD set-up as shown in Figure 1. Even though a separation of energy supply and feed supply is achieved thereby, the FGMD configuration still suffers from the same drawbacks as the DCMD arrangement regarding module internal heat recovery, sensitivity towards vapour pressure reduction and the need for an extra heat exchanger to recover the heat from the permeate channel outlet to preheat the feed. Thus, in order to fulfill all three goals listed in the introduction a further modification is needed. FGAGMD describes a variant in which not only the feed solution is separated from the heating stream, but the cooling stream is also separated from the permeate channel. Such a channel arrangement has previously been discussed in conjunction with MD process integration into heat exchangers by [56,57], but no full scale modules are available to the knowledge of the authors. Due to the addition of the extra second polymer film an additional conductive heat transfer resistance is added to the thermodynamic resistance chain. Also, it must be noted that an amount of sensible heat is transported out of the system together with the heated feed solution in the FGMD and FGAGMD channel configurations and is dependent on the feed solution flow rate, physical properties and temperature. This must be accounted for in the design of the overall system design of a potential FGMD or FGAGMD module and system, if this exiting heat is to be recovered. Due to the higher suitability of an air gap configuration for the concentration of solutions with a high vapour pressure reduction compared to pure H_2_O and the advantages regarding the recovery of heat inside the module, a FGAGMD channel configuration was chosen for the pilot system and developed within the HighCon project. In addition, goals II and III can only be achieved with a separation of heat supply and feed supply to the module(s). Further advantages of the configuration are worth mentioning. The heating and cooling loop can be operated with a single load of water and easily connected directly to available heating and cooling sources without interfering with the feed stream. 

### 2.2. Module Design

The goals of MD module design can be outlined as packaging the process into a device that facilitates a uniform flow distribution in the channels, and reduces energy demand, pressure losses and polarization effects. Cleaning, maintenance and replacement of components should be possible at a reasonable cost. Packing density should be high. All materials used must be mechanically, chemically and thermally adequate for the targeted application of the module [28].

**Figure 2:**
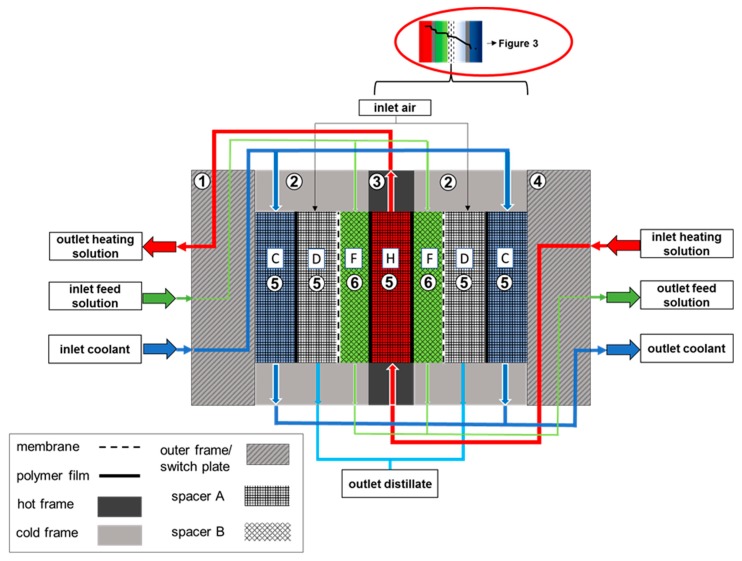
Detailed schematic overview of the FGAGMD channel configuration.

**Figure 3:**
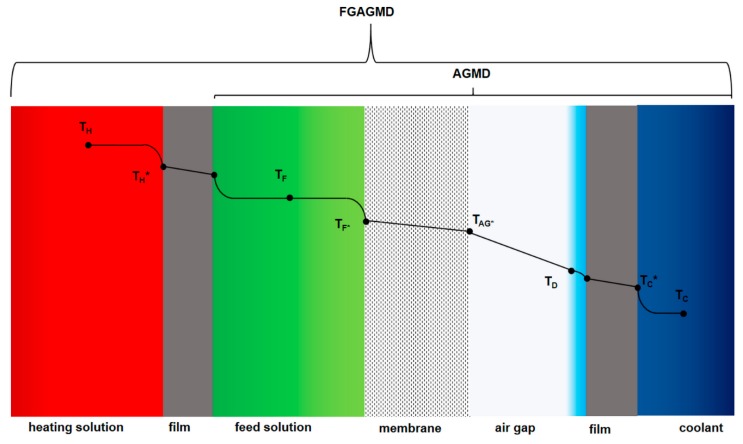
Temperature progression along the cross-section of the channels.

A plate and frame FGAGMD module was designed, with an effective channel length of 5.76 m and channel height of 0.72 m. The channel length in an MD module with sensible heat recovery determines the temperature difference which will be established across the membrane. In opposition to a spiral wound module, the possible channel lengths in a plate and frame type are limited by the length of the individual plates that constitute the channel. The effective channel length can only be a multiple of the single plate length by a serial connection of plates. The single plates are hydraulically connected to each other via switch plates in order to achieve a 180° angle change in flow direction at the end of each plate. This way thermodynamic adaptations to specific customer needs can be implemented easily by adding or subtracting a certain number and arrangement of plates. Figure 2 provides a detailed overview of how the FGAGMD channel configuration was implemented in the prototype module. In order to achieve a minimal heat loss to the ambient, the channels on the respective outer sides of the module were designed to be cold channels. Thus, the only way to package the channel configuration, is to locate the hot channel in the middle of the individual channel set-up. In the schematic in Figure 2, the channels are indicated by the letters H (heating channel), C (cooling channel), D (distillate) and F (feed). The membrane is between each feed (F) and distillate channel (D) and the heating and feed as well as the cooling and distillate channels are separated by a thin polypropylene film. The numbers in the channels and plates of the schematic give reference to the respective material properties in Table 1. Distillate permeation from the feed channels (F) to the distillate channels (D) occurs due to driving force temperature difference established between the heating channel (H) and the cooling channel (C). The basic thermodynamic principles of MD are well published [9,40,58,59] and will not be repeated in detail here. The evaluation of the new module concept will be carried out with use of common key performance indicators presented in Section 2.4. However, the differences of the new FGAGMD to a standard AGMD configuration should be briefly explained. As presented in Figure 3, in FGAGMD an additional conductive resistance is added through the extra heating channel and polymer film. In AGMD the heating channel is equivalent to the feed channel. The second decisive difference is the heat carried in and out of the system with the feed channel. Depending on the operation parameters, sensible heat leaves the system with the outlet of the feed solution. One method to recover this heat is shown in the experimental set-up of the pilot system explained in Section 2.3.

**Figure 4:**
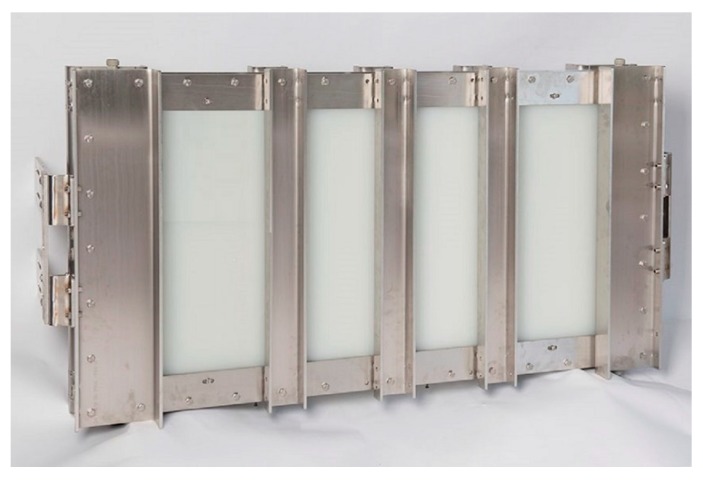
Plate and frame FGMD module.

A closer look at the temperature progression from the hot channel to the right-side cold channel is shown in Figure 3. As indicated in Table 1, the thickness of the heating channel is twice that of the cooling channel since the capacities of the total heating and cooling flow should be as similar as possible for the sake of process efficiency as shown by [38]. The schema also shows how the FGAGMD set up adds to the loss of effective temperature driving force difference at the membrane interface. The loss is expressed by the difference between T_H*_ and T_F*_ and can be assigned directly to the conductive heat loss through the additional film between the feed and the heating solution as well as the additional thermal boundary layers in the feed solution. An assembled module is shown in Figure 4. Including the outer reinforcements, the dimensions of the module are 1 m × 2 m and the inner channel dimensions of a single plate are 0.72 m × 1.44 m. The membrane area of the individual modules is ~8 m^2^. This module′s shell was designed to hold a much higher membrane surface than implemented in this pilot system of up to 50 m^2^. By parallelizing multiple channels, the packing density can thus be improved significantly. 

### 2.3. Experimental Set-Up

**Figure 5:**
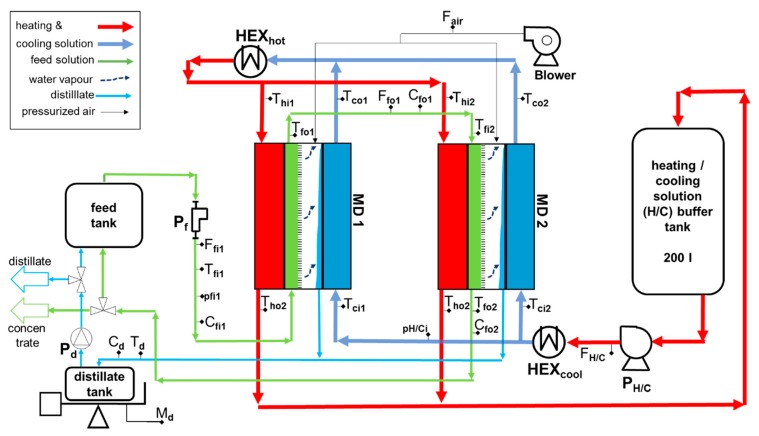
Schematic of the FGAGMD pilot system.

The pilot system consists of an FGAGMD module pair and a hydraulic system and heating on board. The modules are identical twins. It is important to note that only system components were considered, that are freely available in the market and declared as suitable for the application by the manufacturer. The heating and cooling loop is operated in parallel, however, the direction of feed flow is serial. As visible in Figure 5, the feed flow in module 1 is in parallel to the cooling channel whereas in module 2 it is parallel to the heating channel. This enables the recovery of the sensible heat leaving MD 1 in the second module MD 2. For a better understanding of the pilot set-up the flows and components shown in Figure 5 are described as follows: 

#### 2.3.1. Feed Loop

Using feed pump *P*_f_, feed solution with the properties recorded by sensors *T*_fi1_ (temperature feed in MD 1), *p*_fi1_ (pressure feed in MD 1) and *C*_fi1_ (conductivity feed in MD 1) and a volume flow set by flow meter *F*_fi1_ is pumped from the feed tank into the first module (MD 1). After entering, the temperature of the feed solution is increased by the heating solution in a counter current manner through the thin polymer film. Thus, a thermal driving force is established between the feed solution and the cooling channel, which results in a vapour flux passing from the feed solution to the adjacent distillate channel. The remainder of the feed solution (flow *F*_fo1_), leaves MD 1 at temperature *T*_fo1_ and at a conductivity of *C*_fo1_ and enters MD 2 at temperature *T*_fi2_ which is lower than *T*_fo1_ due to small losses to the ambient. Similarly, in MD 2 a vapour flux to the distillate channel is established via the temperature-induced vapour pressure difference. However, in MD 2 the feed channel flow is co- current to the heating channel. It exits MD 2 at temperature *T*_fo2_ and conductivity *C*_fo2_ and flows either back into the feed solution tank or leaves the system depending on the position of the control valve *V*_f_. It is notable that the feed stream is not actively heated or cooled by any sources other than the MD modules. This means, that besides the feed tank, feed pump, piping and sensors, no other components require resistance towards highly concentrated salt solutions (or potentially other chemicals).

#### 2.3.2. Heating and Cooling Loop

The heating and cooling (H/C) solution is pumped from the H/C solution buffer tank with pump *P*_HC_ controlled by flow meter *F*_HC_. No specific materials need be used here, since the heat transfer fluid consists of softened tap water. In case of a leakage, a system shut-down would be triggered to protect the non-salt water resistant components in this loop. Similarly, pressure sensor *p*_HCi_ has a safety function. It protects the modules from overpressure but also provides a recorded value for the monitoring of pressure during dynamic operations. Before entering both modules in parallel, the H/C solution is cooled to a set temperature via heat exchanger HEX_cool_. *T*_ci1_ and *T*_ci2_ record the respective cooling channel inlet temperatures of MD 1 and 2 of which *T*_ci1_ is the control sensor. The cooling channels are separated from the distillate channels by a thin polymer film. The coolant gains temperature due to the recovery of heat from the combination of conductive heat transfer and the latent heat of the distillate condensing on the film. At the outlet, the temperatures are recorded by *T*_co1_ and *T*_co2_. The required external heat is supplied by HEX_hot_ after which the cooling solution becomes the heating solution by definition. In the pilot plant, the thermal energy is supplied by a heating rod and controlled to a fixed temperature monitored by sensor *T*_hi1._ After the heating and cooling solution has entered the module, its temperature reduces constantly along the channel due to the heat transfer through the hot side polymer film in order to provide the process heat to the feed. It exits the heating channel at temperatures *T*_ho1_ and *T*_ho2_, before flowing back to the H/C buffer tank. 

#### 2.3.3. Distillate

**Figure 6:**
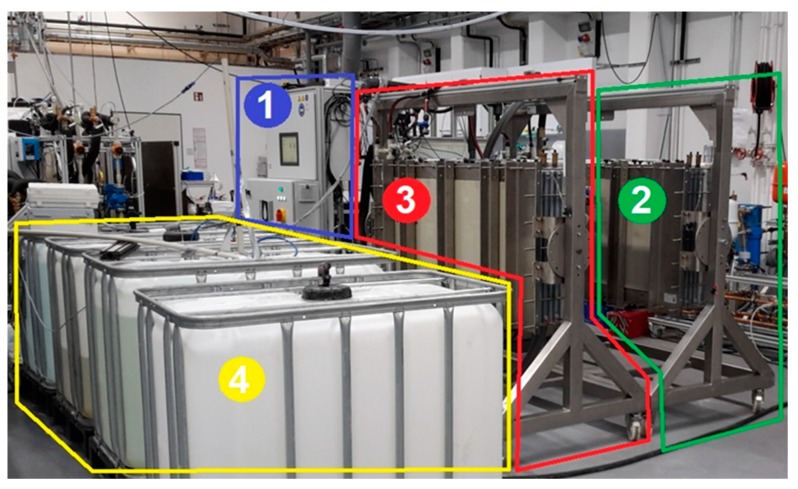
Perspective view of the pilot system and modules; (**1**): rig with pumps, valves and controls, (**2**): module 1; (**3**): module 2; (**4**): feed, concentrate and distillate tanks.

The diffusing water vapour enters the distillate channel “D” through the membrane and condenses on the surface of the condenser channel. The distillate drains either through gravity or is actively drained by low pressurized air supplied by a blower [49]. The volume flow rate of the air is recorded by sensor *F*_air_. The distillate leaves both modules at a mixed temperature *T*_d_ and a conductivity *C*_d_ and is collected in the distillate tank. The tank mass is continuously recorded by the balance *M*_d_. At a set mass value, pump *P*_d_ is triggered and the distillate is pumped either back to the feed tank, for operation continuous mode or discharged to the CIP tank to be used for the periodic cleaning of the module alongside other purposes. The CIP system is not shown in Figure 5 as its details are not relevant to this work, other than the fact that flushing of the module is possible in a fully automatic manner. Figure 6 shows an image of the entire pilot system with indication of the main components.

Permanent logging of all sensor values took place during operation to allow a high resolution of data points and, therefore, an adequate accuracy in data analysis. The logging frequency was set to 30 s per datapoint. Table 2 provides a complete overview of sensors and actors used in the system alongside their accuracy (if applicable) as indicated by the manufacturer.

### 2.4. Key Performance Indicators 

For evaluation of the MD process characteristics and comparison with other modules and systems, well-established key performance indicators (KPI) will be introduced in the following and used in this work. 

Physical properties of salt solutions differ from pure H_2_O. Artificial NaCl–H_2_O solutions at different salinities were used for the characterisation of the new module type. Thus, a series of measurements was carried out to allow a conversion of the online measured and logged conductivity values σ (mS/cm) into salinity values S (g/kg). Formulas (1) and (2) were derived for different concentration ranges.

(1) $$S_{\left(5<\sigma <219.5\right)}=0.0017\sigma^{2}+0.4309\sigma +5.5678$$

(2) $$S_{\left(\sigma >219.5\right)}=0.0392\sigma^{2}+16.1099\sigma +1830.2558$$

The temperature and salinity dependent values for density and specific heat capacity of NaCl–H_2_O solutions were derived from the correlations provided by [60].

The weight measurement of the distillate *M*_d_ (see Figure 5) over time, allows the calculation of the distillate flow rate ${\dot{m}}_{d}$. 

From that, the transmembrane Flux $j_{d}$ (kg/m^2^ h) in Equation (3) can be calculated. Here, the distillate flow rate ${\dot{m}}_{d}$ is put in relation to the active membrane surface A. Flux is well established in all membrane technologies and therefore allows a good comparison between them regarding the specific production of distillate/permeate.

(3) $$j_{d}=\;\frac{{\dot{m}}_{d}}{A}\left[\frac{\mathrm{kg}}{\left(m^{2}h\right)}\right]$$

Another important KPI is the GOR which shows the relation between the thermal energy amount needed for the pure evaporation process of the distillate (numerator) and the amount of heat introduced externally (denominator).

(4) $$GOR=\;\frac{{\dot{m}}_{d}*\Delta h_{v}}{{\dot{m}}_{H/C}*c_{p}*\left(T_{\mathrm{hi}}-T_{\mathrm{co}}\right)}\left[-\right]$$

wherein $h_{v}$ (kJ/kg) is the specific evaporation enthalpy, ${\dot{m}}_{H/C}$ (kg/h) is the mass flow rate of the heating and cooling solution, $c_{p,H/C}$ (kJ/kgK) the specific heat capacity the heating and cooling solution, $T_{\mathrm{hi}}$ the heating inlet temperature and $T_{\mathrm{co}}$ the cooling outlet temperature. The GOR can be calculated separately for each module by using the matching in- and outlet temperatures or in total for the module pair using the total distillate production of both together with the total heat supplied to the modules. 

Within the experiments, distillate mass flow is recorded via a weight value by a balance. Feed flow however, is recorded as a volume flow ${\dot{v}}_{f}$. Since the physical properties of NaCl–H_2_O solutions change with increasing salinity, the calculation of:

(5) $$\dot{m_{f}}={\dot{v}}_{f}*\rho_{f}\left[\frac{\mathrm{kg}}{h}\right]$$

was carried out with density values determined at the respective feed concentration and 25 °C (temperature at point of volume flow measurement) according to [60]. Based on the same reference, specific heat capacity values $c_{p}$ were also determined for the respective concentrations for the mean process temperature $T_{m}$ in the MD module calculated as:

(6) $$T_{m}=\;\frac{\left(T_{\mathrm{ci}}+T_{\mathrm{co}}+T_{\mathrm{hi}}+T_{\mathrm{ho}}\right)}{4}\;[\text{°}C]$$

wherein $T_{\mathrm{ci}}$ is the temperature at the cooling inlet, $T_{\mathrm{co}}$ the temperature at the cooling outlet and $T_{\mathrm{hi}}$ and $T_{\mathrm{ho}}$ are the temperatures at heating inlet and outlet respectively. $T_{m}$ was typically between 51–52 °C within the experiments conducted for this work. 

Thermal efficiency $\eta_{\mathrm{th}}$ given in Equation (7) describes the percentage share of latent heat for the liquid vapour phase change of distillate in relation to the total heat transported through the membrane.

(7) $$\eta_{\mathrm{th}}=\frac{{\dot{m}}_{d}\cdot h_{v}}{{\dot{m}}_{H/C}\cdot c_{p,H/C}\cdot \left(T_{\mathrm{hi}}-T_{\mathrm{ho}}\right)}\cdot 100\;\left[\%\right]$$

Recovery ratio (RR) is the ratio of distillate mass flow rate to H/C flow rate:

(8) $$RR=\frac{{\dot{m}}_{d}}{{\dot{m}}_{f}}\cdot 100\;\left[\%\right]$$

In Section 3.3, a new factor *R*_F_ is introduced which describes the ratio of heating and cooling flow to feed flow:

(9) $$R_{F}=\;\frac{{\dot{m}}_{H/C}}{{\dot{m}}_{f}}[-]$$

## 3. Results

### 3.1. Performance Characterization with Tap Water

In order to evaluate and compare the performance of the new module type with the help of KPIs defined in the previous section, measurements and parameter variations with tap water were carried out. The influence of NaCl–H_2_O solution on the performance is presented in Section 3.2. The coupling of the module pair, with respect to the serial connection of the feed channels, leads to different performance and temperature profiles in MD 1 and MD 2. The main reason for this is the introduction of additional heat into MD 2, transported by the preheated feed leaving MD 1. Thus, the individual module performance profiles will be analyzed at the beginning of this section, but subsequent evaluation will be carried out for MD 1 and MD 2 combined as a pair, displaying mean values. Volume flow of the heating and cooling loop is given in L/h since it is tap water in all cases and the physical properties, especially density, can be assumed to be constant and equal to 1 kg/L. Regarding the feed flow values, mass flow values were derived from the measured volume flows using physical properties of NaCl solution [60]. The conductivity of the distillate produced was below 19 µS/cm during the entire testing regime with tap water. The pilot system was operated for a total of ~21 weeks with interruptions for the moving of the system from the first pilot site in Berlin to the second site in Freiburg, Germany.

**Figure 7:**
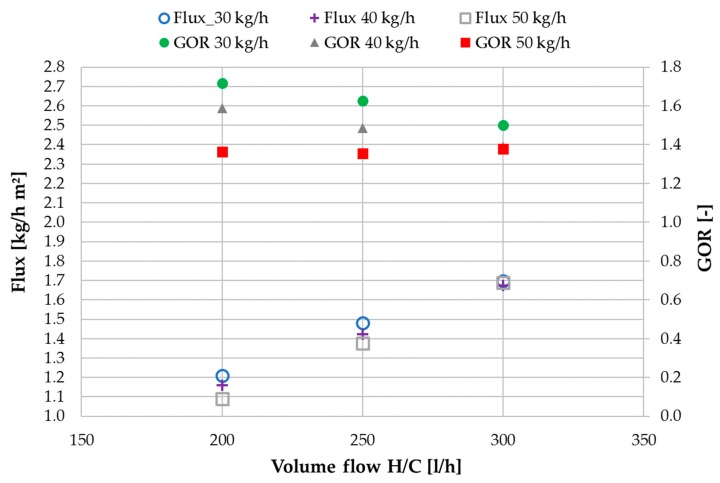
MD 1: influence of H/C volume flow on flux and GOR values at different feed flow rates; tap water; *T*_hi ½_ = 80 °C, *T*_ci ½_ = 25.

Figure 7 shows the influence of heating and cooling volume flow on flux and GOR in MD 1. For better legibility, flux is depicted on the left and GOR on the right y-axis. Flux increases proportionally with the increase in H/C volume for all three feed mass flows as a result of the higher thermal energy input into the system and subsequent increase in bulk temperature difference. This correlation is a well-known characteristic of the membrane distillation process. The flux values are not very sensitive towards the variation of feed mass flow. However, a certain impact can be observed at 200 and 250 L/h H/C volume flow. When increasing the feed flow, more heat is lost for MD 1 through the heated feed stream. Thus, at lower H/C flow rates at which the overall driving force is lower, the impact of that effect is higher, resulting in lower flux for higher feed mass flow. GOR values for 40 and 50 kg/h of feed follow the well- known trend of reducing with increased H/C volume flow. This is due to that fact, that the increase in driving force temperature difference is not completely compensated by the increase in flux. For a feed mass flow of 50 kg/h the succession of GOR values show that the driving force has been reduced over proportionally by the additional heat requirement to heat the entering feed flow. At 300 L/h H/C flow, the GOR untypically increases slightly due to the now sufficient driving force supplied by the H/C loop. At this point it could be deducted that the feed flow rate should be as low as possible, in order to maximize efficiency. For a single module set up, this conclusion would be valid as long as the saturation level of the feed is not reached within the channel after distillate extraction. As mentioned earlier, in a double module set-up the heat exiting MD 1 with the feed, is recovered in MD 2 by introducing it into the hot side of the module. The feed stream exits MD 1 at approximately the mean temperature between *T*_hi1_ and *T*_co1_ (~69–71 °C) depending on the respective flow profile. Small losses occur through the piping between the two modules. Feed temperature was ~68–70 °C on entering MD 2. The total distillate output of MD 1 was ~10–14 kg/h depending on the flow profile. This corresponds with the flux multiplied by the membrane area of 8 m^2^ per module. 

Figure 7 shows flux and GOR values for MD 2. It must be pointed out, that the feed mass flows entering MD 2 are lower than indicated in the naming 30, 40 and 50 kg/h. These values indicate the mass flow at the inlet of MD 1. Due to the distillate extracted in MD 1, they reach MD 2 reduced by the respective output for each operation point. Table 3 shows the feed gap mass flow values for each H/C flow and FG flow combination tested within the tap water characterization measurements. 

**Figure 8:**
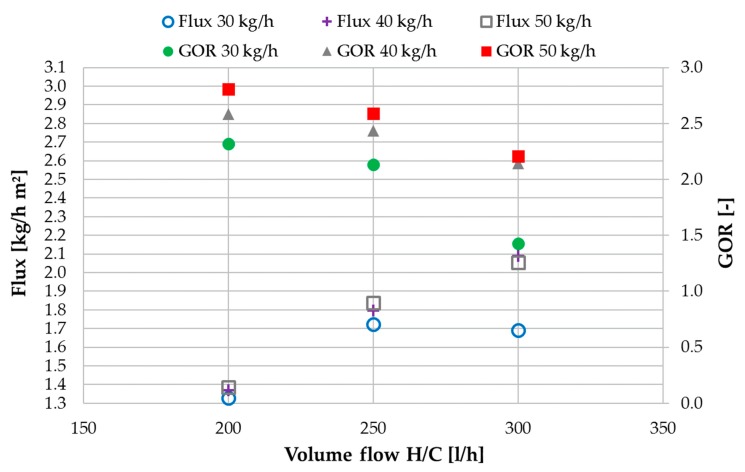
MD 2: influence of H/C volume flow on flux and GOR values at different feed flow rates; tap water; *T*_hi ½_ = 80 °C, *T*_ci ½_ = 25.

There are some major differences in the flux and GOR values of MD 2 which are shown in Figure 8. To begin with, the overall higher flux values, are a direct result of the additional thermal energy being supplied to MD 2 through the preheated feed coming from the outlet MD 1. In MD 2 the flux is largely insensitive to variations of the feed flow with exception of 30 kg/h. At 300 L/h H/C flow and 30 kg/h FG inlet flow, the flux is only 1.7 kg/h m² which is equal to the value at a 250 L/h H/C flow. This phenomenon can be explained as follows: In this case the distillate output is limited by the available feed mass flow entering MD 2. Since the flux is the highest at 300 L/h H/C flow and 30 kg/h FG flow in MD 1, not enough feed is transferred to MD 2 for the total possible flux to be produced. As shown in Table 3, 16.3 kg/h are fed to MD 2 which is the lowest FG in MD 2 value overall. Only 2.7 kg/h of feed leave MD 2. Since this phenomenon does not occur at a FG inlet flow of 40 kg/h, it can be concluded that the optimal feed flow for maximized flux at an H/C flow of 600 L/h lies in between 30 and 40 kg/h. The corresponding GOR value is also significantly reduced for that specific operational point since GOR is calculated in relation to the mass of distillate produced. 

In opposition to MD 1, in MD 2 the GOR values are the highest for the highest feed flow of 50 kg/h in addition to be being overall higher than the values in MD 1. At a feed flow of 50 kg/h, the GOR is ~3 in MD 2 and ~2 in MD 1 at the same H/C flow of 200 L/h. In analogy to the increased flux, the reason lies in the additional heat supply entering MD 2 through the preheated feed from MD 1. It increases the cooling flow outlet temperature *T*_co2_, thus decreasing the delta T (dTh) on the hot side of MD 2. In correspondence with Equation (4) a higher flux or output in the numerator and a lower temperature difference between *T*_hi_ and *T*_co_ in the denominator will lead to a higher GOR. This effect increases, the higher the feed flow to MD 2 is, due to the increase in thermal capacity of the flow. 

**Figure 9:**
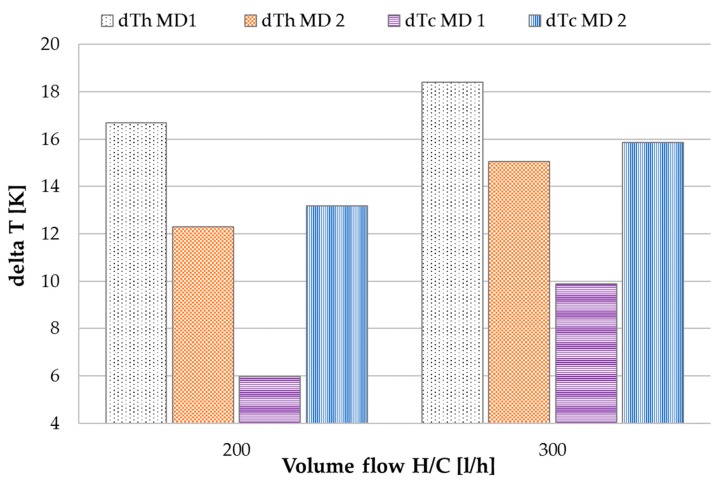
Influence of H/C volume flow on temperature differences in MD 1 and MD 2; ${\dot{v}}_{f}$ = 40 kg/h, *T*_hi ½_ = 80 °C, *T*_ci ½_ = 25.

It has been mentioned that the temperature profiles of MD 1 and MD 2 are affected by the feed flow in different degrees. Figure 9 shows the temperature differences dTh on the hot and dTc on the cold side of MD 1 and MD 2 at different H/C volume flows. dTc is the difference between *T*_ho_ and *T*_ci_ of the respective module. *T*_hi ½_ was set at 80 °C and *T*_ci ½_ at 25 °C. The comparison of dT values in Figure 9 is carried out for a feed inlet flow of 40 kg/h. The dTh MD 1 values are higher than then dTc MD 2 values for both the given flow rates. Heat leaves the system with the feed outlet on the hot side of MD 1 leading to a decrease in heat recovery in the module and subsequently a lower *T*_co1_. Thus, dTh MD 1 is higher than dT MD 2 in which that heat is added to the thermal energy supply coming from the heat exchanger HEX_hot_. *T*_co2_ increases, resulting in the lower dTh MD 2 values depicted in the bar chart. Since the heating inlet temperature *T*_hi_ is set at 80 °C, the generally increased delta T hot values at 300 L/h are result of decrease in cooling outlet temperature *T*_co_. The hot side delta T ratio of MD 1–MD 2 decreases from 4.4 to 3.3 K. Because a higher flux is generated at this flow rate, the feed flow reaching MD 2 is lower than at 200 L/h. This results in a disproportional relation of the hot side delta Ts to one another when the H/C flow rate is increased at the same feed inlet mass flow rate. Opposite effects can be observed regarding the cold side delta Ts. dTc is much lower in MD 1 than in MD 2. On the cold side of the module, *T*_ho1_ is decreased by the additional thermal capacity of the feed entering MD 1 at approximately the same temperature as *T*_ci_. By the time the feed has reached the outlet of MD 2, the mass flow has so far reduced, that the impact on *T*_ho2_ is low. This is shown by the fact that dTh MD 2 and dTc MD 2 are very similar as could be expected e.g., in a counter current heat exchanger. dTh MD 2 is 0.9 K lower than dTc MD 2 at 200 L/h and 0.8 K lower at 300 L/h H/C flow. It has been shown by [38] that a symmetrical temperature profile along the flow channels of MD modules will have a beneficial effect on process efficiency. This can be achieved by synchronizing the mass flow capacities in the flow channels. In this new channel configuration however, complexity is added by the mass flow capacity of the feed-baring channel. In MD 1, the capacity of the feed channel ads to that of the cooling channel due to their co-current flow relation. In MD 2 however, the feed flows co-current to the heating channel making the temperature profiles in MD 1 and MD 2 not only asymmetrical but also unequal, however, with a much lower impact in MD 2 due to the lower feed mass flow. A reduction in process efficiency must be expected in comparison to a configuration with parallelized temperature profiles as a trade-off for the advantages of being able to establish a one- step process design. 

**Figure 10:**
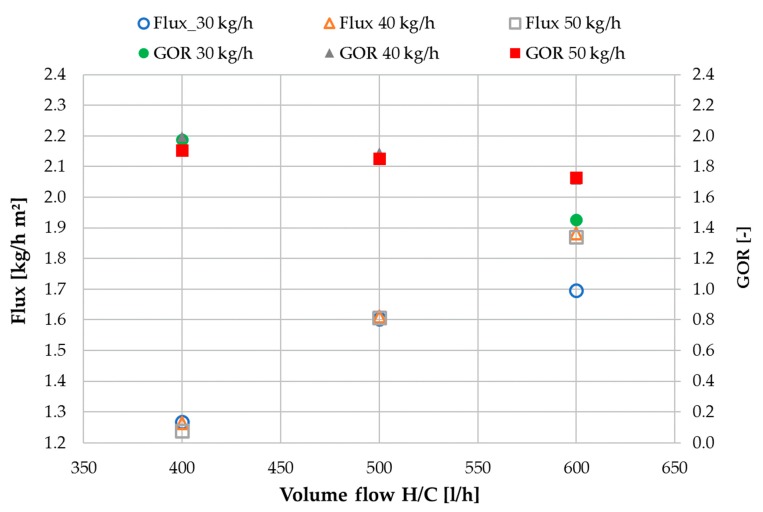
Influence of HC volume flow on Flux and GOR values at varied feed flow rates; MD 1 + MD 2 combined; Tap water; *T*_hi ½_ = 80 °C, *T*_ci ½_ = 25.

Figure 10 shows mean flux and GOR values for MD 1 and MD 2 combined. Total output was recorded and then divided by the total membrane area of both modules to derive flux. Similarly, GOR was calculated by taking into account the total output and the total heat used for both modules. Total H/C flow for both modules is depicted on the x-axis. It can be observed, that when considering MD 1 and MD 2 as a joined concept, the difference between 40 and 50 kg/h feed flow rate is no longer visible since all sensible heat leaving MD 1 with the feed flow is recovered in MD 2. This is however not valid for 30 kg/h for reasons explained in conjunction with Figure 8. All of the following graphs show combined values for MD 1 and MD 2. 

**Figure 11:**
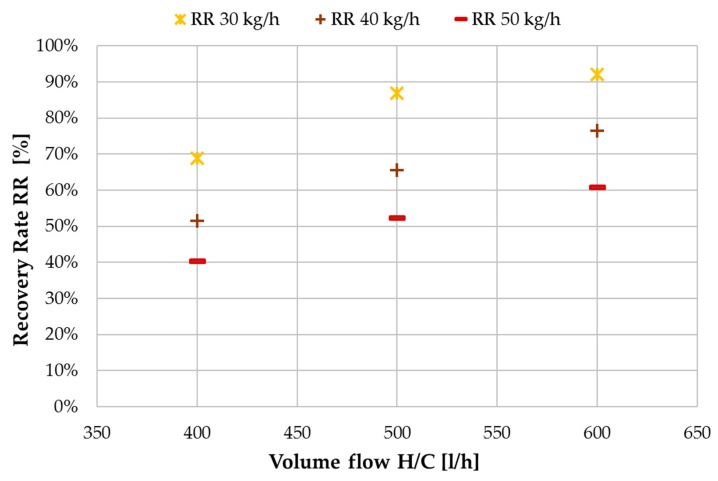
Influence of H/C volume flow on recovery ratio (RR) at different feed flow rates; MD 1 + MD 2 combined; tap water; *T*_hi ½_ = 80 °C, *T*_ci ½_ = 25.

One of the main reasons for separating the heating and cooling flow from the feed flow, is the possibility of controlling the recovery ratio of the module independently of the energy supply. Figure 11 shows the achieved recovery ratios with tap water as feed. Since the recovery ratio RR is a ratio of distillate output to feed input (Equation (8)), more distillate production from the same feed flow rate will result in a higher RR. Thus, with increase of H/C flow the RR values also increase. As shown in Figure 10, the distillate flux does not change significantly with changes in feed flow rate. This results in a reduction of RR for increasing feed mass flow. It should be pointed out, that the highest value of 93% RR was achieved at a H/C flow rate of 600 L/h and feed flow of 30 kg/h. This value might even be exceedable if the aforementioned limitations regarding distillate production in MD 2 had not occurred. 

**Figure 12:**
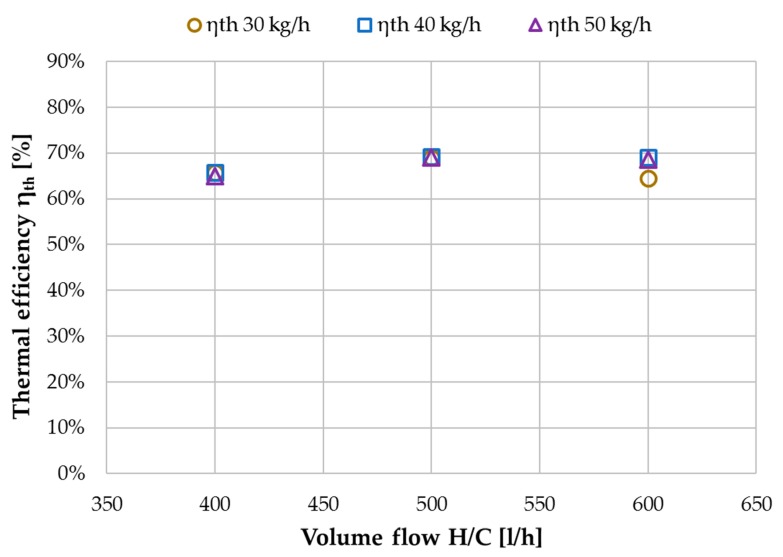
Influence of H/C volume flow on thermal efficiency η_th_ at different feed flow rates; MD 1 + MD 2 combined, tap water, *T*_hi ½_ = 80 °C, *T*_ci ½_ = 25.

Thermal efficiency η_th_ (Equation (7)) indicates the fraction of latent heat in relation to the total heat transported through the membrane. Since the increase in flux is proportionate to the increase in H/C flow, the η_th_ values presented in Figure 12 remain approximately constant for H/C flow variation. η_th_ will serve as a KPI in the comparison with a previously analyzed spiral wound module in Section 3.4. Since Figure 12 shows mean values for both modules, it is worth mentioning that the individual values were not identical but within ~10% of each other. The tap water characterization presented within this section has shown, that the proposed concept of a FGAGMD channel configuration with a double module strategy provided the desired operational behavior. In the new concept, the basic KPIs flux GOR, recovery ratio RR and thermal efficiency η_th_. showed the same dependencies as expected in membrane distillation with a very high increase in achievable recovery rate.

### 3.2. Performance Characterization with NaCl Solution

Membrane distillation is capable of concentrating solutions to near saturation, provided that crystal formation does not occur. Being an ambient pressure process, limitations due to e.g., a high osmotic potential of the solution are not an issue. In MD, however, thermal energy is required to bring about the evaporation of the feed solution. With an increasing amount of salt ions in the feed solution, the required energy to evaporate the same amount of distillate increases, due to the decrease in vapour pressure of the solution. For a given MD system with defined channel geometry and fixed operation conditions, this means that flux and GOR will decrease continuously, the more salt ions are present in the feed solution. It is challenging to achieve a high GOR in hypersaline brine concentration with MD. Published data with full-scale modules is scarce but of highest importance for assessment of the real performance of MD technology. Testing and piloting result such as those presented in this work cannot and should not be compared to results achieved with tap water or measured in small scale lab equipment. 

**Figure 13:**
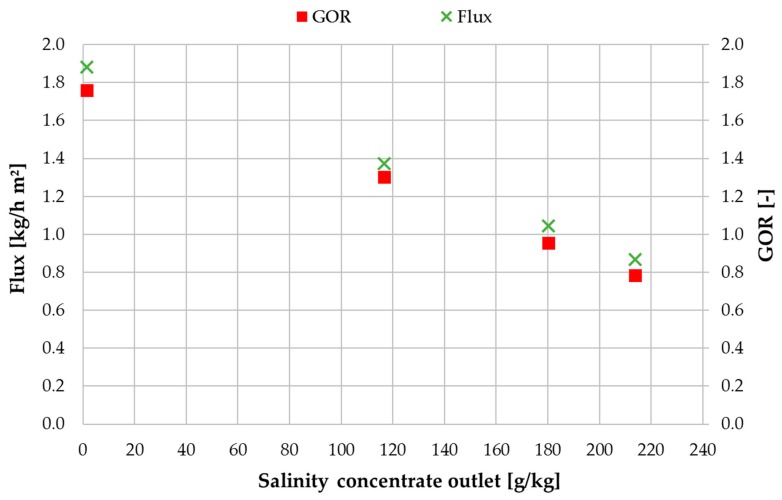
Influence of concentrate salinity on flux and GOR; MD 1 + MD 2 combined; feed inlet salinity 0.3/55/95/143 g NaCl/kg; ${\dot{v}}_{f}$ = 40 kg/h, ${\dot{v}}_{H/C}\;$= 600 L/h, *T*_hi ½_ = 80 °C, *T*_ci ½_ = 25.

Figure 13 shows the value trend of flux in correlation with concentrate salinity at an H/C flow rate of 600 L/h and an inlet feed flow rate of 40 kg/h. The salinity of the concentrate at the outlet of MD 2 was selected for depiction on the x-axis, flux and GOR are shown on the left and right y-axis respectively. During these experiments the blower, indicated in Figure 5, was operated continuously at an avg. air volume flowrate of 17 L/h. It has been shown by [49] in a study with an AGMD module, that when operating with salt solutions, the benefit of optimal draining of the distillate in the air gap channel has a significant positive impact on the distillate quality. A low-pressure air blower is an efficient method of improving the draining. The distillate conductivity was below an avg. of 1 mS/cm during all the salt solution measurements. The well-known impact of vapour pressure reduction [39] can be observed distinctly in Figure 13. Beginning at ~1.9 kg/h for the lowest salinity, flux reduces to 0.87 kg/h for the highest outlet salinity of 214 g NaCl/kg. The succession of GOR values is corresponding to this and provides a range of 1.76–0.78 depending on the concentrate salinity. 

**Figure 14:**
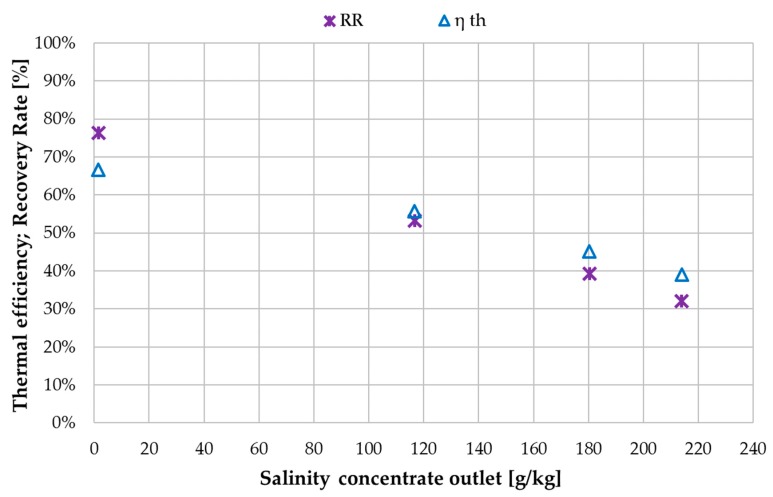
Influence of concentrate salinity on recovery ratio (RR) and thermal efficiency η_th_; MD 1 + MD 2 combined; Feed inlet salinity 0.3/55/95/143 g NaCl/kg; ${\dot{v}}_{f}$ = 40 kg/h, ${\dot{v}}_{H/C}$ = 600 L/h, *T*_hi ½_ = 80 °C, *T*_ci ½_ = 25.

The decrease in thermal efficiency ηth over salinity is shown in Figure 14. The reduction in vapour pressure caused by the increase in dissolved ions in the solution reduces the effective driving force for evaporation. Thus, the ratio of heat used for actual phase change in relation to the total heat transferred through the membrane and air gap shifts with increasing feed salinity. With tap water, 67% of the total heat is being used for evaporation. At 214 g NaCl/kg this fraction is reduced to 39%. The recovery ratio is furthermore affected by the reduction in flux. At the lowest salinity of 1 g NaCl/kg, 76% of the feed going into the inlet of MD 1 is extracted as distillate. This means that 9.6 kg/h exits MD 2 out of the 40 kg/h which entered MD 1. At 214 g NaCl/kg, the recovery ratio RR reduced to 32% as a result of the reduced flux. 

### 3.3. Ratio of H/C Solution Flow to Feed Flow

**Figure 15:**
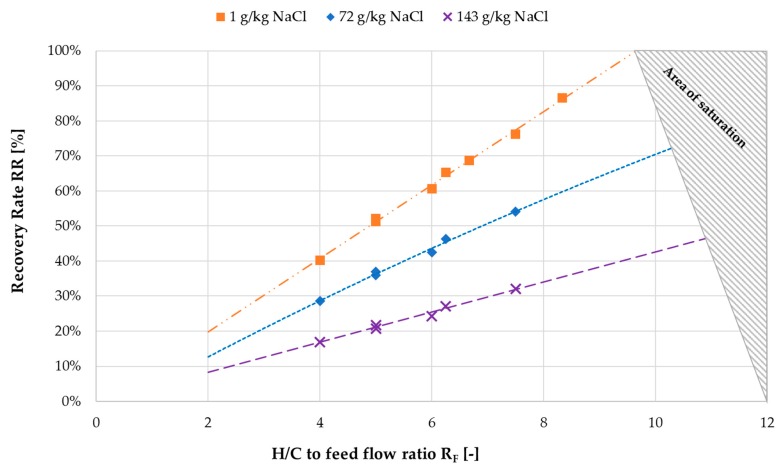
Effect of H/C flow to feed flow ratio *R*_F_ on recovery ratio (RR); *T*_hi ½_ = 80 °C, *T*_ci ½_ = 25.

For a deeper understanding of the benefits of the overall module and system concept some key facts should be elaborated more closely. The goal of the concept is to achieve the desired increase in feed solution concentration in a single pass process. This was not achieved in this particular prototype system due to a restriction in the systems operation. The H/C flowrate was limited to 600 L/h, due to the capacity of pump *P*_H/C_. As established by the evaluation of the tap water characterization measurements, the ratio of heating and cooling flow rate to feed flow rate has a high impact on the recovery ratio. The effect is connected to the proportionally higher flux production out of the same amount of feed flow when increasing the H/C flow rate. Thus, a new ratio *R*_F_ is established (Equation (9)), which describes the quotient of H/C mass flow ${\dot{m}}_{H/C\;}$ to feed mass flow ${\dot{m}}_{f}$. Figure 15 depicts the influence of *R*_F_ on recovery ratio RR for three different feed salinity levels. The impact of both *R*_F_ and salinity on recovery ratio can now be observed. Linear fit curves are used to show the progression of the recovery ratio with increasing *R*_F_. The required *R*_F_ for a desired recovery ratio increases with increasing feed salinity. For example, for a required RR of 50% in a single pass process at an inlet feed concentration of 1 g NaCl/kg, *R*_F_ of ~5 would be sufficient according to the operating temperatures of the pilot system. At 143 g NaCl/kg inlet concentration however, the inclination of the values is much lower and a higher *R*_F_ would be required, taking into consideration that NaCl–H_2_O solution is saturated at ~253 g NaCl/kg. These correlations are dependent on the module’s channel length and are only valid for this specific module. Nonetheless, the principles for future module and process design remain equal for any geometry. For a given inlet feed concentration and a desired final concentration, *R*_F_ must be determined in order to achieve the required recovery ratio. Certain boundary conditions must also be considered. In order to sustain overall efficiency, the feed flow cannot be lowered to a value that does not supply a sufficient amount of feed to MD 2. The negative effects on GOR and thermal efficiency were analyzed in Section 3.1. In addition, a safety margin to prevent saturation of the solution in MD 2 should be added to the minimum feed flow rate.

The recommended procedure for selection of feed inlet flow rate and *R*_F_ can be summarized as follows:

- Determination of feed concentration;
- Selection of desired outlet concentration;
- Selection of required *R*_F;_
- Determination of minimum feed flow;
- Calculation of H/C flow rate according to *R*_F._

Depending on the available heat supply, the channel length of the modules will be designed. Channel length selection is a key parameter in MD module design, both technologically as well as economically as explained in detail in [22]. However, the GOR and flux are much more sensitive to channel length modification than the overall output of the module. In consequence, *R*_F_ ratios are not expected to change in a large magnitude for the same temperature profile and channel lengths within 4–9 m. 

### 3.4. Comparison with Spiral Wound Air Gap Membrane Distillation (AGMD) Module

Within this work so far, a novel type of MD configuration was presented and analyzed. The analysis was based upon a first-generation prototype plate and frame module. A comparison with the previously studied spiral wound AGMD module type should nonetheless be conducted at this stage in order to identify any possible disadvantages of the overall concept. Even though the packaging of the modules is different, no significant differences in the influence of operational parameters on the KPIs of the process are expected. Materials and membrane types are identical.

**Figure 16:**
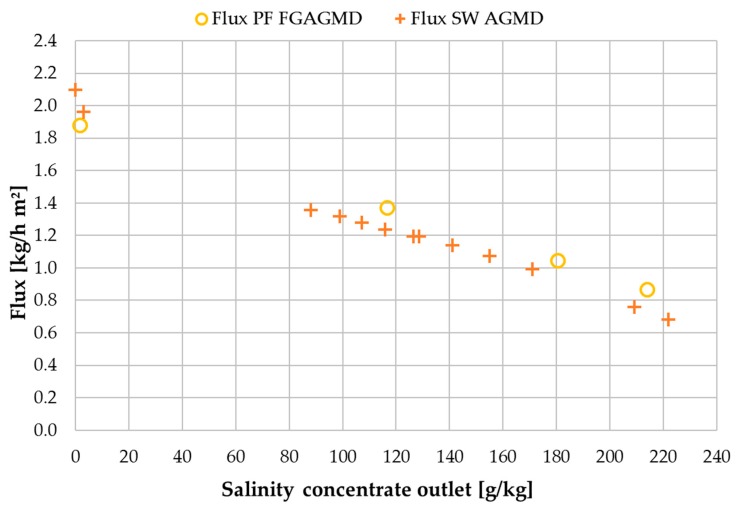
Influence of concentrate salinity on flux in plate and frame FGAGMD and spiral wound AGMD modules; ${\dot{v}}_{f}$ = 40 kg/h, ${\dot{v}}_{H/C}$ = 300 L/h per module; ${\dot{v}}_{fsw}$ = 300 L/h; *T*_hi_ = 80 °C, *T*_ci_ = 25.

**Figure 17:**
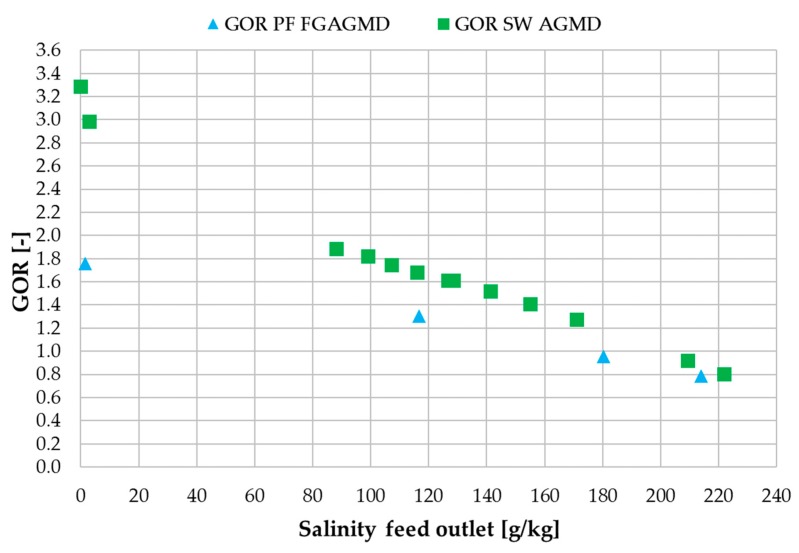
Influence of concentrate salinity on GOR in plate and frame FGAGMD and spiral wound AGMD modules; ${\dot{v}}_{f}$ = 40 kg/h, ${\dot{v}}_{H/C}$ = 300 L/h per module; ${\dot{v}}_{fsw}$ = 300 L/h; *T*_hi_ = 80 °C, *T*_ci_ = 25.

The data used for comparison is extracted from a study with an AGMD spiral wound module and hypersaline brine which can be found with [49]. The AGMD module had a 6 m channel length and was operated with the same flow of 300 L/h. Figure 16 shows flux and Figure 17 shows GOR values of both modules in direct comparison. Flux values for both module types are very similar but with slightly higher values for the spiral wound for tap water and slightly higher values for the plate and frame for at salinities above ~80 g NaCl/kg. This shows that for a similar channel length and under the same operating conditions regarding the heating and cooling flows, the flux is similar regardless of the module type. The internal heat recovery of the modules expressed as GOR, however, shows some differences especially at tap water salinity. This effect can be assigned to the additional heat-transfer resistance added by the feed gap in the plate and frame. In the low-salinity region where vapour pressure depreciation does not have an impact, the larger delta T caused by the additional thermal resistance leads to a higher energy requirement per mass unit of distillate which directly effects the GOR. At the same time, this higher effective delta T leads to advantages over the spiral wound module at higher conductivities since more net driving force is available after subtracting the fraction of driving force lost to the reduction in vapour pressure. The asymmetrical temperature profiles in the FGAGMD module shown in Section 3.1. also account for a reduction in the overall GOR values of this module type.

**Figure 18:**
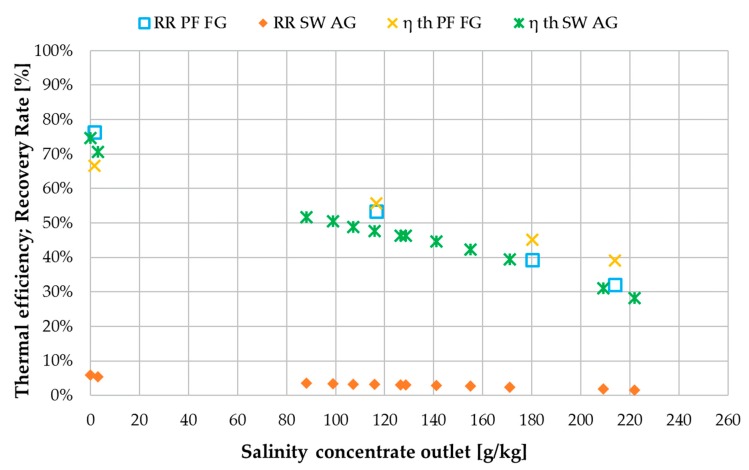
Influence of concentrate salinity on thermal efficiency η_th_ and recovery ratio (RR) in plate and frame and spiral wound module types under similar operating conditions; ${\dot{v}}_{f}$ = 40 kg/h, ${\dot{v}}_{H/C}$ = 300 L/h per module; ${\dot{v}}_{fsw}$ = 300 L/h; *T*_hi_ = 80 °C, *T*_ci_ = 25.

When comparing recovery ratio and thermal efficiency shown in Figure 18, the most significant difference is in the recovery ratio. At tap water the difference is 70% and at 214 g NaCl/kg the difference is still approximately 30%. The enabling of such high recovery ratios was one of the core motivations for the implementation of the new FGAGMD channel configuration. As mentioned previously, the recovery ratio of the spiral wound AGMD module is not independently adjustable due to the coupling of heat supply and feed supply. Thermal efficiency values are similar for both modules with the largest differences at the low end of the salinity range. On average for the entire salinity range tested, however, η_th_ of the FGAGMD module was higher by approximately 4% compared to the spiral wound module.

From the direct comparison of the two module types, it can be deducted that even in this first prototype stage, the plate and frame (PF) FGAGMD module has a general advantage over the spiral wound (SW) AGMD module when implemented in high concentration applications. Under the assumption that e.g., a RO brine at a salinity 7 g NaCl/kg should be concentrated to 240 g NaCl/kg [22], at the resulting avg. salinity of 155 g NaCl/kg the FGAGMD module is be superior in performance regarding flux, η_th_ and RR with only small drawbacks in GOR. Table 4 provides a summary of KPIs at the mentioned avg. salinity of 155 g NaCl/kg. 

Further potential for improvement on the plate and frame module design is definitely given, especially regarding flow distribution and channel geometry. It is expected that similar GOR values to the AGMD module will be possible after optimization of the inner components of the module. Furthermore, an increase in channel length can be considered in order to increase the GOR. This will, however, decrease the flux and require more membrane area. The application of vacuum to the air gap would have a significant positive effect on efficiency, though drawbacks in distillate quality are to be expected when implementing this option.

## 4. Conclusions

Within this work, a novel plate and frame FGAGMD module was presented. By separating the heating and cooling channel from the feed channel, the concept allows a minimum amount of components in contact with the highly corrosive feed. Furthermore, it decoupled the thermal energy supply from the feed supply, giving room for a new range of operational flow settings. The goal of increasing the recovery ratio of the single pass process was achieved with values of up to 93% RR using tap water as feed and between 32–53% with NaCl solutions ranging between 117 and 214 g NaCl/kg. The impact of well-known correlations on the KPIs flux, GOR and η_th_ remained valid for this new module type. For optimization of flow rate strategies for a given range of concentration a new ratio of H/C flow to feed flow was introduced named *R*_F_. *R*_F_ serves as an indicator for the selection of a flow regime to achieve a required recovery ratio. In comparison to a previously analyzed AGMD spiral wound module, the FGAGMD plate and frame prototype showed similar performance characteristics with slight improvements regarding flux and η_th_. As expected, the recovery ratio was between 12–16 times higher in the plate and frame module (32–93%) than in the spiral wound (2–6%) due to the new channel configuration. Drawbacks of the FGAGMD module were observed in GOR especially in the lower salinity region. For an average salinity of 155 mS/cm, however, the difference in GOR reduced approx. 0.3. It is likely that the GOR of the plate and frame module can be improved by optimizing the internal flow distribution within the channels which was not the focus in the construction of this first prototype module. In general, for the applications with corrosive, toxic or otherwise hazardous media the implementation of a FGAGMD plate and frame MD module opens up a new range of applications for the technology with advantages in cleaning and maintenance, safety and the integration into industrial wastewater treatment processes. Further improvements must be carried out to optimize the efficiency and long- term testing will be necessary with a follow-up prototype in order to properly evaluate lifetime cycles of all replacement parts. 

Furthermore, an in depth investigation of the thermodynamics of the FGAGMD process in a flat sheet bench scale testing facility will published in a follow-up publication. This will enable the validation of modelling tools and the optimization of efficiency and operational key factor *R*_F_ with respect to different salinity levels.

## Figures and Tables

**Table 1:** Material properties of sub-components used inside the module.

No	Material	Thickness	Polymer	Approx. Porosity (%)	Nominal Pore Diameter (µm)
1 + 4	Outer- and switch plate	30 mm	Polypropylene (PP)	-	-
2 + 3	Hot and cold channels	4 mm/2 mm	PP	-	-
5	Spacer A	2 mm	High density Polyethylene (HDPE)	80	-
6	Spacer B	1 mm	PP	80	-
-	Membrane (+ backing)	76 (+280) µm	Polytetrafluorethylene (PTFE), (PP)	80 (50)	0.2
-	Polymer film	100 µm	PP	-	-

**Table 2:** Sensor and actor list of the pilot system.

Component	Producer	Type	Accuracy	Name in Schematic
Conductivity meters	Jumo, Fulda, Germany	CTI500 24VDC	≤0.5% of measuring range (0–500 mS/cm)	*C*_fi1_, *C*_fo2_
Conductivity meter distillate	Jumo, Fulda, Germany	BlackLine CR-EC	≤2% of measuring range (0–5000 µS/cm)	*C*_d_
Volume flow H/C	Krohne, Duisburg, Germany	Optiflux 4300C	0.5% of measuring value	*F*_H/C_
Volume flow Feed	MIB GmbH, Breisach am Rhein, Germany	Flowmax 42i	±2% of measured value ± 3mm/s	
Temperature sensors	TC direct, Mönchengladbach, Germany	Pt100 Klasse A	±(0.15 + 0.002 × t)	all Temperatures
Feed pump	KNF Neuberger GmbH, Freiburg, Germany	PML14169-NF 300	-	*P*_f_
Heating and Cooling pump	Dunkermotoren, 79848 Bonndorf im Schwarzwald, Germany	BG 65X50 SI	-	
Pressure sensors	Jumo, Fulda, Germany	Midas, C18 SW -1,6	1.6% of measuring value	*p*_H/Ci_, *p*_fi1_
PLC	Advantech Europe BV, Hilden, Germany	2271G-E1-C20170517	-	-
Pressurized air pump	KNF, Neuberger GmbH, Freiburg, Germany	KNF N828 KNE	-	Blower
Balance distillate	Soehnle Industrial Solutions GmbH, Backnang, Germany	Table balance	1 g for 0–32 kg	*M*_d_
Volume flow air	First Sensor AG, Berlin, Germany	WTA	±2% of reading +0.25% of measuring value	*F*_air_
Heating and cooling pump	Harton Anlagentechnik GmbH, Alsdorf, Germany	DC 40/10BL	-	*P*_H/Ci_

**Table 3:** Feed flow rates at the inlet of MD 1, the inlet of MD 2 and the outlet of MD 2.

H/C	FG in M1	FG in M2	FG out M2
(L/h)	(kg/h)	(kg/h)	(kg/h)
400	30	20.2	9.5
400	40	30.7	19.6
400	50	41.2	30.0
500	30	18.1	4.2
500	40	28.5	14.0
500	50	38.9	24.1
600	30	16.3	2.7
600	40	26.5	9.7
600	50	36.4	19.9

**Table 4:** Average key performance indicator (KPI) values for plate and frame FGAGMD and spiral wound AGMD module, mean concentrate salinity ~155 mS/cm; ${\dot{v}}_{f}$ = 40 kg/h, ${\dot{v}}_{H/C}$ = 300 L/h per module; ${\dot{v}}_{fsw}$ = 300 L/h; *T*_hi_ = 80 °C, *T*_ci_ = 25.

	Flux	GOR	RR	ηth
	(kg/m² h)	(-)	(%)	(%)
PF FGAGMD	1.2	1.1	45	50
SW AGMD	1.1	1.4	3	46

## Nomenclature

A	Membrane area
AGMD	Air gap membrane distillation
C	Conductivity (mS/cm; µS/cm)
cp	Specific heat capacity (kJ/kg K)
DCMD	Direct contact membrane distillation
delta T	Driving force temperature difference (K)
dTh	Temperature difference on the hot side of the module
dTc	Temperature difference on the cold side of the module
F	Flow (L/h)
FGMD	Feed Gap Membrane Distillation
FGAGMD	Feed Gap Air Gap Membrane Distillation
GOR	Gained output ratio ( - )
h	Height (m)
HEX	Heat exchanger
KPI	Key performance indicator
m	Mass flow (kg/h)
MD	Membrane Distillation
MED	Multi Effect Distillation
MSF	Multi Stage Flash
j	Transmembrane flux (kg/m² h)
L	Channel length (m)
p	Pressure (bar)
P	Pump
PP	Polypropylene
PTFE	Polytetrafluorethylene
PGMD	Permeate gap membrane distillation
R	Ratio
RR	Recovery ratio
RO	Reverse Osmosis
S	Salinity (g/kg)
SWRO	Seawater Reverse Osmosis
T	Temperature (°C)
ρ	Density (kg/m³]
η	Efficiency (%)
v-AGMD	Vacuum air gap membrane distillation
v	Volume flow (L/h)
∆hv	Evaporation enthalpy (kJ/kg)

## Indices

ci	cooling inlet
co	cooling outlet
d	distillate
hi	heating inlet
ho	heating outlet
f	feed
F	feed ratio
H/C	heating and cooling
hx	heat exchanger
m	mean
th	thermal
